# Pandemic Influenza as 21st Century Urban Public Health Crisis

**DOI:** 10.3201/eid1512.091232

**Published:** 2009-12

**Authors:** David M. Bell, Isaac B. Weisfuse, Mauricio Hernandez-Avila, Carlos del Rio, Xinia Bustamante, Guenael Rodier

**Affiliations:** Centers for Disease Control and Prevention, Atlanta, Georgia, USA (D.M. Bell); New York City Department of Health and Mental Hygiene, New York, New York, USA (I.B. Weisfuse); Ministry of Health of Mexico, Mexico City, Mexico (M. Hernandez-Avila); Emory University Rollins School of Public Health, Atlanta (C. del Rio); Pan American Health Organization, San Jose, Costa Rica (X. Bustamante); World Health Organization, Geneva, Switzerland (G. Rodier)

**Keywords:** influenza, pandemic (H1N1) 2009, Mexico, United States, emergency medical services, international perspectives, quarantine, viruses, policy review, expedited

## Abstract

The percentage of the world’s population living in urban areas will increase from 50% in 2008 to 70% (4.9 billion) in 2025. Crowded urban areas in developing and industrialized countries are uniquely vulnerable to public health crises and face daunting challenges in surveillance, response, and public communication. The revised International Health Regulations require all countries to have core surveillance and response capacity by 2012. Innovative approaches are needed because traditional local-level strategies may not be easily scalable upward to meet the needs of huge, densely populated cities, especially in developing countries. The responses of Mexico City and New York City to the initial appearance of influenza A pandemic (H1N1) 2009 virus during spring 2009 illustrate some of the new challenges and creative response strategies that will increasingly be needed in cities worldwide.

## Abstract 2

Responses of Mexico City and New York City in spring 2009 illustrate the importance of advance planning.

According to United Nations estimates, the percentage of the world’s population living in urban areas will increase from 50% in 2008 to 70% (4.9 billion persons) in 2025. During 2007–2025, the number of cities with population 1–5 million will increase from 382 to 524, and the number of megacities (>10 million population, comprising the core city, suburbs, and continuously settled commuter areas) will increase from 19 to 27. Of the 27 megacities, 16 will be in Asia, 4 in Latin America, 3 in Africa, 2 in Europe, and 2 in North America. Currently, 1 in 25 persons lives in a megacity; in Latin America, the ratio is 1 in 7. In central Tokyo, the population density is 5,847 persons/km^2^ (*1*). Cities are increasing in developing countries and often have slums that lack basic services (*2*). The accelerating global trend toward megacities is a new paradigm of human existence and poses profound public health challenges. New approaches for surveillance, preparedness, and response will be needed because current strategies may not be easily scalable upward to address huge, densely populated areas, especially in developing countries.

In 2008, the World Health Organization (WHO) International Health Regulations (IHR) Coordination Department, in collaboration with Lyonbiopole (Lyon, France), held a consultation, Cities and Public Health Crises (*1*). Consultants stated that WHO and national guidance does not always adequately address the challenges their cities face, and they could learn much from each other. This article summarizes these challenges, illustrated by the initial appearance of influenza A pandemic (H1N1) 2009 virus during spring 2009 in Mexico City, Mexico, and New York (NYC), New York, USA (metropolitan area populations 20 million and 19 million, respectively). These megacities may not be representative of cities in low-income countries, which face more daunting problems.

## A Tale of 2 Megacities: Pandemic (H1N1) 2009, Spring 2009

### Mexico City

National surveillance detected an atypical increase in influenza-like illness (ILI) in mid to late February 2009 and a further increase in early to mid April. Anecdotal reports in April of increased hospitalizations of previously healthy young adults with severe pneumonia led to active surveillance in 23 hospitals in Mexico City and identification of 47 such cases. Patient samples showed nonsubtypeable influenza A, identified on April 23 as a novel influenza A virus of subtype H1N1. The Mexico City response was based on early adaptation of a pandemic influenza preparedness plan that had been developed for a virus originating abroad. After an expert meeting convened by the secretary of health, given the uncertain potential health impact, the president of Mexico invoked emergency powers; on April 24, community mitigation measures were implemented in Mexico City and the neighboring state of Mexico (*3*–7). These measures were announced and coordinated by the federal government, with participation of state authorities. The objective was to decrease transmission; elements included an intensive mass media campaign to inform the population about influenza, promote personal and environmental hygiene, request that sick persons stay home, and implement social distancing measures. Persons with ILI were encouraged to seek prompt medical care. Early in the epidemic, the federal government released antiviral drugs from the national strategic reserve and controlled their distribution. Ill persons and their close contacts had access to this medication free of charge. During the spring outbreak, an estimated 150,000 cases of ILI with 3,312 hospitalizations occurred in metropolitan Mexico City (H. Lopez-Gatell, pers. comm.).

**Figure 1:**
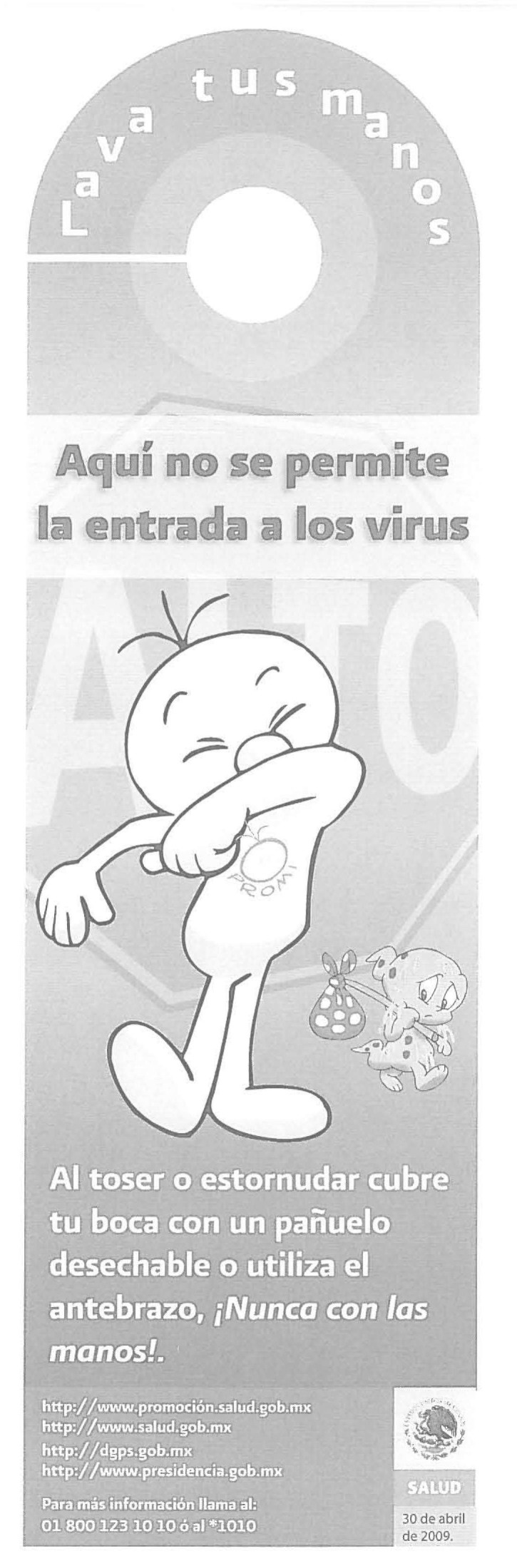
Sign hung on doorknobs containing information from the Mexican Ministry of Health promoting cough etiquette, using the communications icon *Promi* (*3*). Translation: “Wash your hands. Viruses are not permitted to enter here. When coughing or sneezing, cover your mouth with a disposable handkerchief or use your forearm, never your hands!”

Following the Mexican Pandemic Plan, a program of social mobilization was implemented through a multifaceted mass media saturation campaign featuring visual representations and a previously developed and tested message icon, *Promi*, to address Mexico City’s heterogeneous population and literacy rates (Figure 1). The private sector, including pharmacy chains, food stores, and cellular telephone companies, helped deliver health messages. The Mexican telephone company (Telmex) assembled a call center that received >5 million calls. Novel communication strategies included text messaging and mass emails; information from the Ministry of Health was transmitted through >140 million text, 60 million printed, and 18 million email messages. Multilingual health information materials also were provided to all international travelers entering and exiting through Mexican ports, and departing travelers underwent thermal screening.

Frequent hand washing and cough etiquette were promoted, and all government and private facilities open to the public were provided with alcohol gel and other disinfectants. Because of limited water availability in some areas or households, alcohol gel was distributed free. A mass media campaign promoting a healthy distance discouraged greeting others by hugging or kissing, common practice among Mexicans of all social strata. Military personnel distributed disposable surgical masks in public places; their use was recommended primarily for sick persons, but many healthy persons also wore them daily. Compliance with recommendations appeared to be high, although some persons wearing masks may have developed a false sense of security that took priority over cough etiquette and hand washing. When commercially available masks became scarce, some persons made their own, and disposal occasionally was problematic, resulting in littering. Over time, recommendations about cough and sneeze etiquette were followed least frequently.

All educational facilities were closed beginning April 24 in Mexico City and, soon after, nationwide. Parents were advised to keep children at home; authorities distributed educational materials for home use. By May 11, when educational facilities reopened, all schools had been thoroughly cleaned. Parents were requested to keep ill children home; peer pressure among parents to comply was high. Every day, upon arrival at school, children were screened for fever and respiratory symptoms. Ill children were sent home to receive care; return to school required a note from their primary healthcare provider granting medical clearance.

**Figure 2:**
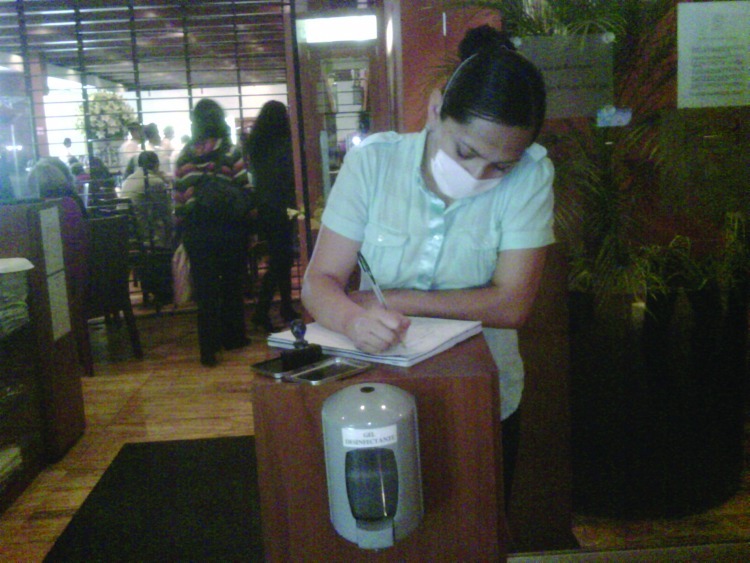
A reopened restaurant in Mexico City, Mexico, illustrating mask use by the person greeting entering customers and a hand hygiene dispenser that all entering customers were required to use, May 2009. Photo courtesy of Carlos del Rio.

In addition to federal measures, on April 27, the mayor of Mexico City suspended dine-in service in all restaurants and similar establishments, allowing only take-out orders. Many restaurants simply remained closed. When affected businesses were allowed to reopen on May 6, social distancing measures (e.g., avoiding crowding) were encouraged, and hygiene measures were enforced (Figure 2). Grocery stores and supermarkets remained open, with additional cashiers used to keep lines short. Persons in public places were advised to remain separated by at least 2 m. Large gatherings were cancelled or postponed, and entertainment venues, e.g., movie theaters, were closed. Professional sports matches were broadcast, but stadiums were closed to the public. Churches and temples also remained closed, with religious services broadcast over radio and television. When normal services resumed, communion cups and other shared objects were wiped with hand gel after each use. Mass transit operated normally. Masks were provided for drivers and passengers and buses and subway cars were cleaned frequently. Mitigation measures were broadly accepted by the public. Occasional early discrepancies between recommendations from official and academic sources (e.g., regarding mask use) led to a few critical media reports without apparent consequence. Thousands of workplaces of all sizes in Mexico City and the rest of the country were closed for several days, taking a huge toll on the economy. The government provided no financial compensation to businesses or workers. The economic impact of pandemic (H1N1) 2009 virus in Mexico during the spring is estimated as >$2.3 billion (0.3% of gross domestic product) (*8*).

Most important among the many lessons learned in Mexico is that preparation paid off. Although requiring adaptation, the preexisting pandemic plan and planning process facilitated intersectoral work, decision making, and rapid development of a public communications campaign. The availability of a national stockpile of antiviral drugs reassured the public. The participation of the secretary of health as the spokesman demonstrated high-level leadership. Clear and transparent communication was important because Mexico was entering mid-term elections, and some politicians hypothesized that the outbreak was a farce to distract Mexicans.

The outbreak also enabled detection of some weaknesses in the Mexican health system. In Mexico, healthcare is provided by 3 major healthcare systems; thus compilation of epidemiologic information regarding hospitalizations was complex. However, after a few days, a system was devised that provided the necessary consolidated information. Laboratory capacity was inadequate for the challenges posed by the outbreak. At the onset of the outbreak, the Ministry of Health had no state-level laboratories with capabilities for influenza molecular diagnostics; all molecular diagnosis was centralized at the National Epidemiological Reference Laboratory in Mexico City. The Ministry of Health rapidly improved the national laboratory network and Mexico has now 28 laboratories (1 in nearly every state) with PCR molecular diagnostic capabilities. Although having a pandemic plan was useful, operationalizaton of the plan was less smooth. For example, procedures existed to close schools, but criteria for reopening them and the ability to reassure parents that reopened schools were safe did not.

### NYC

Emergency preparedness planning in NYC accelerated after the World Trade Center and anthrax attacks of 2001 and in anticipation of an influenza pandemic. Novel syndromic surveillance systems monitor visits to hospital emergency departments, calls to emergency medical services, pharmacy sales, worker absenteeism, and outpatient clinic visits. For example, information is collected electronically for ≈90% of daily patient visits from 77% of emergency departments. Patients’ age, sex, home postal code, and chief complaint, but not names, are transmitted daily to the NYC Department of Health and Mental Hygiene, where protocols identify and follow up signals that suggest increased community illness. During spring 2009, these systems were essential for real-time monitoring of the pandemic in NYC, e.g., documenting large increases in children with ILI seeking care at emergency departments) and for tracking its spread throughout the city from 1 school where it apparently was first introduced and amplified (*9*). Additional systems collect etiologic information from, for example, virologic studies on samples of outpatients and hospitalized patients with ILI. However, the first indication to the health department of the outbreak of pandemic (H1N1) 2009 virus came from a school nurse telephoning a report of increased ILI at a single school. Subsequent surveillance and telephone surveys indicated ≈750,000–1 million persons in NYC had ILI during the spring outbreak (*10*).

When the first cases were confirmed, an extensive public communication campaign was implemented through Ready New York, a preexisting program of the NYC Office of Emergency Preparedness (*11*). The program includes outreach to ethnic populations and translation into many languages. The principal messages were 1) wash hands thoroughly and frequently with soap and water; 2) avoid contact with persons who are obviously sick; and 3) if you get sick with any cold or influenza, stay home from work or school, and avoid contact with others as much as possible

During the epidemic peak, the mayor and health commissioner held frequent press conferences in English and Spanish. A NYC government information hotline (311) previously had been established and featured live operators 24/7, with 98% of calls answered within 30 seconds. During the spring outbreak of pandemic (H1N1) 2009, ≈54,000 calls to 311 about influenza and a smaller health department hotline were answered. An electronic health alert network and conference calls provided messages to healthcare providers.

Aside from the public messages, community mitigation measures focused on selective closure of schools. Household contacts of case-patients were not quarantined, businesses were not closed, and public gatherings were not cancelled unless they involved closed schools. School closures were decided on an individual basis (known as “reactive” closures, based on visits for ILI to the school health nurse and on other factors, such as the ability of students to comply with respiratory hygiene) rather than “preemptively” (i.e., before cases in the school but with reports of cases in other schools in the subdistrict or district). Approximately 50 schools closed, for ≈1 week each.

The NYC emergency stockpile of antiviral drugs was not used because normal distribution channels sufficed. Occasional reports of spot shortages required rapid investigation and highlighted the need for close communication with private distributors. If the stockpile had been needed, antiviral drugs would have been distributed to community health centers, public clinics, and hospitals.

Distribution of vaccine for pandemic (H1N1) 2009 in NYC will depend on indications for use, availability, and urgency of administration. Vaccine will be prioritized for high-risk populations (*12*). Mass vaccination campaigns will use 200 point-of-distribution sites developed to meet possible needs for anthrax prophylaxis, e.g., school buildings throughout the city that each could serve ≈40,000 persons.

Problems included basing decisions on a pandemic severity index because, at the pandemic onset, its case-fatality ratio was uncertain. Despite previous planning, several school dismissal issues had never been entirely resolved, including the objectives of closure in a less severe pandemic (i.e., to protect high-risk students, all students, families; to slow community transmission; to allay public fears). The effectiveness of school closure in meeting these objectives was uncertain, as was the extent to which benefits justified the secondary impact, including interrupting the academic program, parental work loss, and disruption of services provided at school (e.g., free breakfast and lunch to children from low-income families, therapy for students with special needs).

Operational questions included criteria for school dismissal and reopening and difficulties in monitoring ILI and even absenteeism rates among students. Absenteeism data were often unavailable to the health department until mid-afternoon, relatively late to notify parents about closure decisions for the next school day. Instructions were not given for children to remain at home, and some may have recongregated elsewhere, such as in public libraries, while their parents were at work (*13*). News of school closures in NYC led to questions from parents in suburban jurisdictions about why their schools remained open, even though they had no known cases. Individual school closings showed the interconnectedness between schools, such as when siblings or neighbors attended different ones. The issue of worker or business compensation for lost time from work to care for ill children remains difficult. On the basis of this experience, in the 2009–10 school year, NYC is urging parents to keep sick children home and emphasizing infection control at school but will close a school only as a last resort. Closure decisions will be made on an individual basis, taking into account whether infection control practices could be improved and whether a high percentage of students have high-risk medical conditions (*10*).

During spring 2009, emergency departments were overcrowded with the worried ill, despite many announcements about indications for persons with ILI to seek medical care. In the fall and winter of 2009 hospitals are prepared to open additional nonemergency ILI care sites (e.g., at primary care clinics). A new 1-stop influenza Web portal provides information, as well as locations of clinical sites, and a call center staffed by nurses accessed through the 311 hotline provides guidance to persons with ILI (*10*).

In the city jail, cases of pandemic (H1N1) 2009 led to screening and control measures. These included isolation and cohorting of ill prisoners, and quarantine of those who had been exposed to them, to limit the spread of infection in the prison and court systems.

Health department staffing to meet surge needs posed challenges, including accessing and training staff from other parts of the health department, especially physicians, and the need to ensure staff time off to prevent burnout. Keeping policies and press releases consistent in the face of changing science and policies required constant attention. Internet survey instruments were effectively used to collect epidemiologic data, as in the initial high school student outbreak (*9*).

## Issues for Cities

The experiences from the response to the emergence of pandemic (H1N1) 2009 virus in Mexico City and NYC highlighted several challenges raised at the WHO consultation (*1*). These include response coordination, surveillance and monitoring of illness trends, disease containment and mitigation, delivery of countermeasures, and public communication.

### Response Coordination

Multiple government agencies serve large urban areas. Citizens frequently live, work, attend school in, and commute through different jurisdictions. Different political parties may control national, state or provincial, suburban, and city governments. Fringe groups or gangs may effectively control some areas. Incident or unified command systems can be useful approaches to crisis coordination (*14**,**15*). In Mexico City and NYC, advance planning, political leadership at the highest levels, and collaboration among public health and emergency management agencies were particularly important.

Coordination with the private sector often is not well established. Businesses can assist, notably by providing health messages and enabling infectious workers to remain home (*16*). Large companies may have contacts with city leaders, but most are small to medium-sized enterprises with which coordination may be difficult. Multinational corporations, common in cities, may be subject to home-country influences. Response coordination with nongovernment, community, and faith-based organizations also is important. Outbreaks in cities near international borders require coordination with foreign partners.

### Surveillance and Monitoring of Illness Trends

Emerging challenges in cities include the vertical dimension (high-rise apartment blocks), travelers, and persons who do not have fixed addresses or who live in slums. Novel approaches for illness reporting and population surveys may include use of cell phones and the Internet. Illness surveillance ultimately depends on the organization and provision of health services; cities with universal health coverage will have important advantages (*17*). Outbreak recognition still often depends on alert clinicians; technology-based systems notwithstanding, a school nurse provided the first indication of the influenza outbreak in NYC.

### Disease Containment and Mitigation

Although not generally a problem in Mexico City or NYC during spring 2009, in a larger outbreak in a lower-income country, home isolation or quarantine may be difficult or impossible for large urban families living in 1 or 2 rooms. Contact tracing is problematic in cities, given the frequency of anonymous interactions. Innovative use of nonhealth databases and 3-dimensional mapping, including cell phone records and global positioning technologies, may be helpful but may pose privacy issues.

Decisions regarding school dismissal are problematic because effectiveness for disease mitigation is difficult to quantify, and operational aspects often are uncertain, whereas the potential for societal disruption is considerable. Analysis is pending of the different approaches taken by Mexico City and NYC during spring 2009, but both have kept schools open during the fall, because pandemic severity has remained comparable with that in the spring. This approach is consistent with updated guidance from WHO and the US Centers for Disease Control and Prevention (*18**,**19*). Many questions remain about how to implement social distancing and infection control measures in typical city venues, including schools, institutions of higher education, healthcare institutions, mass transit, workplaces, and marketplaces. These issues are even more difficult in developing countries. Many cities have international airports and may need to assist in health screening of passengers; provide medical care to ill passengers; and accommodate stranded passengers, including those in quarantine. Evacuation of a city poses additional public health challenges (*17**,**20*).

### Delivery of Countermeasures

Rapid delivery of countermeasures, e.g., drugs and vaccines, is difficult even for persons with known, fixed addresses, but more so for persons in slums, travelers, undocumented persons, and homeless persons, as well as the elderly and homebound. Measures taken in Mexico City and NYC during spring 2009 appear to have been sufficient, but these systems are being tested again during the 2009–10 winter season.

### Public Communication

WHO outbreak communication guidelines emphasize building and maintaining trust, announcing information early, ensuring transparency, listening to the public, and planning ahead (*21*). The generally successful public communication campaigns of Mexico City and NYC incorporated these approaches. In addition to traditional mass media and the Internet, they also used cell phones and text messaging, which may offer useful models for developing countries. Cell phone networks may need to prioritize health or emergency messages, improved robustness to permit high traffic during emergencies, and redundancy in case transmitting towers are destroyed (e.g., in a storm). Text messages can be targeted geographically, e.g., to phones locked on to a particular transmitting tower at the time of the message. This approach could be useful for broadcasting localized alerts and instructions, such as locations of vaccination clinics.

## Discussion

Cities are the norm of global development in the 21st century. As cities become larger and more crowded, traditional guidance for detecting and responding to public health crises requires innovation. Modified guidance may be helpful, but new strategies, technologies, and metrics also will be needed.

Preliminary accounts of response to pandemic (H1N1) 2009 during spring 2009 in 2 world megacities offer grounds for optimism. In each case, advance planning laid the foundation for enhanced surveillance and a generally effective response, made possible by an extensive public communications campaign and effective political leadership. On the other hand, challenges emerged that would have been amplified if the illness had been more severe or the period of societal disruption prolonged. Development of new guidance and approaches requires collaboration among large cities, as well as research and evaluation to identify best practices for cities with different resource levels, particularly for implementing core capacity requirements under the revised IHR in a world where most persons now live in urban environments. The IHR require all countries to have core capacity for disease “surveillance, reporting, notification, verification, response and collaboration activities” by 2012 (*22**,**23*). These requirements must be implemented in urban environments, but they are based on traditional public health levels (local, intermediate, and national), which are less clearly defined for large urban agglomerations. All national governments have committed themselves to IHR implementation; municipalities must play a central role but may not be aware of their obligations or able to meet them. Many partners will be important, including businesses, which may not realize their stake in IHR implementation (*24*). Sharing of experience and research is needed to develop strategies and best practices that can be considered by similar cities worldwide.
